# Impact of the Novel Prophage ϕSA169 on Persistent Methicillin-Resistant Staphylococcus aureus Endovascular Infection

**DOI:** 10.1128/mSystems.00178-20

**Published:** 2020-06-30

**Authors:** Liang Li, Genzhu Wang, Yi Li, Patrice Francois, Arnold S. Bayer, Liang Chen, Kati Seidl, Ambrose Cheung, Yan Q. Xiong

**Affiliations:** a The Lundquist Institute for Biomedical Innovation at Harbor-UCLA Medical Center, Torrance, California, USA; b The Genomic Research Laboratory in Geneva, Geneva, Switzerland; c David Geffen School of Medicine at UCLA, Los Angeles, California, USA; d Department of Medicine, Division of Infectious Diseases, Harbor-UCLA Medical Center, Torrance, California, USA; e Center for Discovery and Innovation, Nutley, New Jersey, USA; f University Hospital of Zurich, Zurich, Switzerland; g Dartmouth Medical School, Hanover, New Hampshire, USA; Vanderbilt University Medical Center

**Keywords:** MRSA, bacteriophage, persistent endovascular infection

## Abstract

Persistent methicillin-resistant Staphylococcus aureus (MRSA) endovascular infections are life-threatening syndromes with few therapeutic options. The potential impact of bacteriophages on the persistent outcome has not been well studied. In this study, we investigated the role of a novel prophage (ϕSA169) in MRSA persistence by using a lysogen-free clinically resolving bacteremia (RB) isolate and comparing it to a derivative which was obtained by infecting the RB strain with ϕSA169, which has been lysogenized in a clinical persistent MRSA bacteremia (PB) isolate. Similar to the PB isolate, the ϕSA169-lysogenized RB strain exhibited well-defined *in vitro* and *in vivo* phenotypic and genotypic signatures related to the persistent outcome, including earlier activation of global regulators (i.e., *sigB*, *sarA*, *agr RNAIII*, and *sae*); higher expression of a critical purine biosynthesis gene, *purF*; and higher growth rates accompanied by lower ATP levels and vancomycin (VAN) susceptibility and stronger δ-hemolysin and biofilm formation versus its isogenic parental RB isolate. Notably, the contribution of ϕSA169 in persistent outcome with VAN treatment was confirmed in an experimental infective endocarditis model. Taken together, these results indicate the critical role of the prophage ϕSA169 in persistent MRSA endovascular infections. Further studies are needed to identify the mechanisms of ϕSA169 in mediating the persistence, as well as establishing the scope of impact, of this prophage in other PB strains.

**IMPORTANCE** Bacteriophages are viruses that invade the bacterial host, disrupt bacterial metabolism, and cause the bacterium to lyse. Because of its remarkable antibacterial activity and unique advantages over antibiotics, for instance, bacteriophage is specific for one species of bacteria and resistance to phage is less common than resistance to antibiotics. Indeed, bacteriophage therapy for treating infections due to multidrug-resistant pathogens in humans has become a research hot spot. However, it is also worth considering that bacteriophages are transferable and could cotransfer host chromosomal genes, e.g., virulence and antimicrobial resistance genes, while lysogenizing and integrating into the bacterial chromosome (prophage), thus playing a role in bacterial evolution and virulence. In the current study, we identified a novel prophage, ϕSA169, from a clinical persistent MRSA bacteremia isolate, and we determined that ϕSA169 mediated well-defined *in vitro* and *in vivo* phenotypic and genotypic signatures related to the persistent outcome, which may represent a unique and important persistent mechanism(s).

## Abstract (precis)

Bacteriophages are viruses that invade the bacterial host, disrupt bacterial metabolism, and cause the bacterium to lyse. Because of its remarkable antibacterial activity and unique advantages over antibiotics, for instance, bacteriophage is specific for one species of bacteria and resistance to phage is less common than resistance to antibiotics. Indeed, bacteriophage therapy for treating infections due to multidrug-resistant pathogens in humans has become a research hot spot. However, it is also worth considering that bacteriophages are transferable and could cotransfer host chromosomal genes, e.g., virulence and antimicrobial resistance genes, while lysogenizing and integrating into the bacterial chromosome (prophage), thus playing a role in bacterial evolution and virulence. In the current study, we identified a novel prophage, ϕSA169, from a clinical persistent MRSA bacteremia isolate, and we determined that ϕSA169 mediated well-defined *in vitro* and *in vivo* phenotypic and genotypic signatures related to the persistent outcome, which may represent a unique and important persistent mechanism(s).

## INTRODUCTION

Methicillin-resistant Staphylococcus aureus (MRSA) is a leading cause of life-threatening endovascular infections, especially bacteremia and infective endocarditis (IE) (1, 2). Persistent MRSA bacteremia (PB; defined as ≥7 days of positive blood cultures in the presence of antibiotic therapy) represents ∼15 to 30% of such infections (3, 4). Most PB strains appear to be susceptible *in vitro* to standard-of-care anti-MRSA antibiotics (e.g., vancomycin [VAN] and daptomycin [DAP]) by CLSI breakpoints, yet persist *in vivo* despite seemingly appropriate antibiotic therapy (4–6). This paradox has fostered a number of investigations in our laboratories focused on a specific molecular mechanism(s) that underlies the *in vivo* persistent outcome.

Prophage elements have been demonstrated to contribute to the pathogenesis of staphylococcal infections (7–9). In some cases, prophages can impact bacterial fitness and host-microbe interactions, genetically correlating with well-defined immunomodulatory virulence factors (e.g., *lukF-PVL*) (7, 10), as well as staphylococcal global regulators (e.g., *sigB*) (9). Importantly, there is growing evidence suggesting prophages might also be involved in bacterial persistence due to their effect on promoting biofilm formation and triggering the stringent response, which is a metabolic signaling pathway activated by nutritional stresses (8, 9).

**FIG 1:**
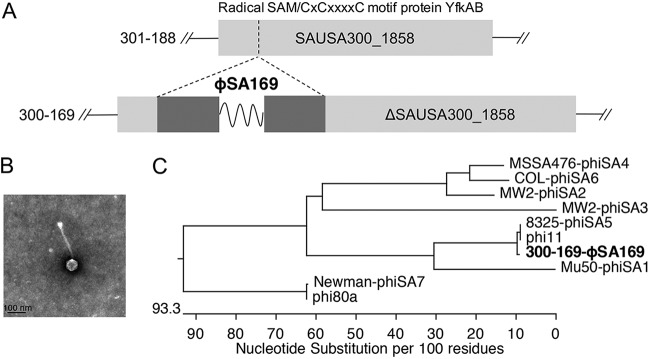
General characterizations of bacteriophage ϕSA169. (A) Location of the prophage ϕSA169 in the SAUSA300_1858 sequence in the 300-169 (JASL01000001) chromosome and the corresponding location in the 301-188 (JASK01000004) chromosome. (B) Transmission electron microscopy image of viable ϕSA169 particle. (C) Classification of ϕSA169 based on integrase serogroup.

Our recent whole-genome sequence (WGS) analyses identified a novel temperate prophage, ϕSA169, in a prototypical clinical PB strain (300-169) but not present in a genetic-background-matched (clonal complex, *agr*, and SCC*mec* types) RB strain (301-188; RB defined as initial MRSA bacteremia resolved within 2 to 4 days of antibiotic therapy) (11). More specifically, the WGS data demonstrated that ϕSA169 was inserted into a chromosomal gene, SAUSA300_1858 (encoding protein YfkAB), in the PB strain; this gene was uninterrupted in the RB strain (Fig. 1A). A transmission electron microscopy image of a viable ϕSA169 particle is presented in Fig. 1B. The genome of prophage ϕSA169 has a size of ∼44 kb, shows ∼79% similarity to the genome of phage ϕ11, and can be grouped with ϕSA5 by integrase homology (Fig. 1C) (11, 12). Although the phenotypic and genotypic distinctions between PB and RB strains have been well described previously (5, 13), the causal interaction between ϕSA169 and the persistent outcome has not yet been studied. Thus, the current investigation was aimed to reveal the role of prophage ϕSA169 in the persistence of MRSA endovascular infection.

## RESULTS

### Successful transduction of ϕSA169.

**FIG 2:**
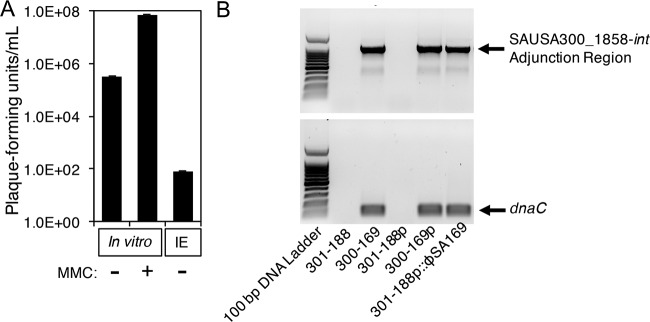
(A) Titers of ϕSA169 in the recipient strain RN4220 after exposure to the supernatant of filtered 300-169 stationary-phase cells with and without MMC exposure and plasma from 300-169-infected rabbits. (B) PCR amplification of SAUSA300_1858-*int* adjunction region and the phage gene *dnaC* of 300-188, 300-169, 301-188p, 300-169p, and 301-188p::ϕSA169 strains.

The titers of ϕSA169 in the recipient strain, RN4220, after exposure to the supernatant of filtered 300-169 stationary-phase cultures with/without mitomycin C (MMC) exposure and plasma from 300-169-infected rabbits with IE were 7 × 10^7^ PFU/ml, 3.3 × 10^5^ PFU/ml, and 80 PFU/ml, respectively (Fig. 2A). Similar results were observed following the transduction of ϕSA169 into the RB 301-188 strain. For instance, the titer of ϕSA169 in RB 300-169p with MMC exposure was 3 × 10^7^ PFU/ml. PCR results indicated that all study strains, except 301-188 and 301-188p, contained the SAUSA300_1858-*int* adjunction region and the phage gene *dnaC* (Fig. 2B). These results suggest that ϕSA169 from the donor strain (PB 300-169p) was successfully integrated into the SAUSA300_1858 gene in the recipient RB strain 301-188.

### WGS further confirmed the transduction of ϕSA169 in the RB 301-188 strain.

The WGS data indicated that both 300-169p and 301-188p::ϕSA169 contained identical genomic DNA sequences of ϕSA169 (see Fig. S1 in the supplemental material). ϕSA169 integrated into the SAUSA300_1858 gene in the RB 301-188p strain at the same position as in the genome of the PB 300-169p strain (Fig. S1). Of the sequences other than that of ϕSA169 and the plasmid pALC1766, there were only 3 single-nucleotide polymorphisms (SNPs) demonstrated among 301-188, 301-188p, and 301-188p::ϕSA169 (Table S1). Of these three SNPs, one synonymous mutation was found in the gene encoding a hypothetical protein in 301-188p::ϕSA169, compared to 301-188 and 301-188p. The other two were identified in genes encoding a siderophore biosynthesis protein and phosphoenolpyruvate carboxykinase in 301-188p versus 301-188, suggesting that these two SNPs might have occurred during the generation of 301-188 carrying pALC1766 (13) and have no substantial impacts on PB-related phenotypes (Table S1).

10.1128/mSystems.00178-20.1 FIG S1 Linear comparison of prophage and surrounding regions in S. aureus strains 300-169, 300-188, and 301-188p::ϕSA169. Download FIG S1, DOCX file, 0.4 MB. Copyright © 2020 Li et al. 2020 Li et al. This content is distributed under the terms of the Creative Commons Attribution 4.0 International license.

10.1128/mSystems.00178-20.2 TABLE S1 Characteristics of single-nucleotide polymorphisms in study S. aureus strains. Download Table S1, DOCX file, 0.01 MB. Copyright © 2020 Li et al. 2020 Li et al. This content is distributed under the terms of the Creative Commons Attribution 4.0 International license.

### Transduction of ϕSA169 converted the RB strain into a strain with PB-like phenotypes.

**FIG 3:**
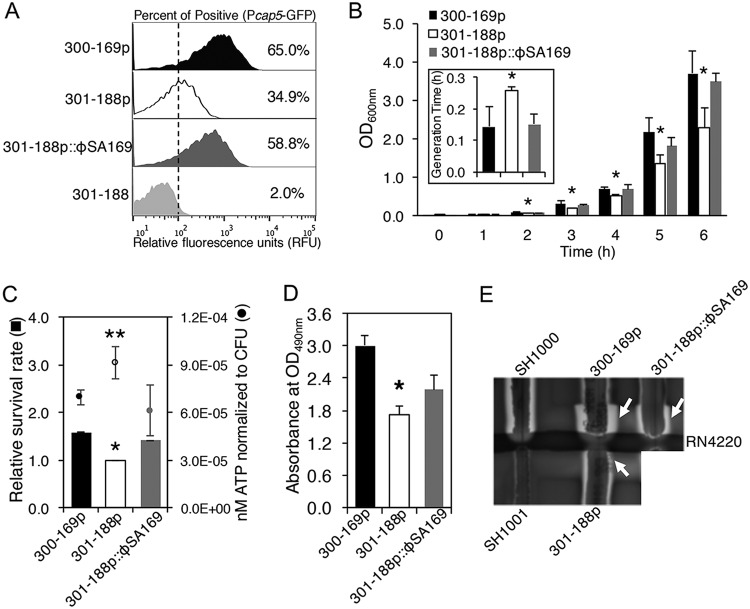
Expression of *cap5* in 300-169p, 301-188p, and 301-188p::ϕSA169 strains at 6-h incubation time by flow cytometry (A). Relative fluorescence units (RFU) were used to measure green fluorescent protein (GFP) expression. Cells with >10 RFU were demonstrated as positive for GFP expression. The results are representative of flow cytometry data in histograms from one replicate (A). Growth curves and generation times (inset; during exponential phase) (B), ATP levels at late-exponential phase (6-h incubation) and relative survival rates to VAN (C), biofilm formation (D), and δ-hemolysin activity (E) in 300-169p, 301-188p, and 301-188p::ϕSA169 strains. *, *P* < 0.01; **, *P* < 0.001, versus 301-188p strain.

The correlation among *cap5* promoter activation, bacterial growth, ATP levels, and VAN susceptibility has been well elucidated (13, 14). For example, our previous studies indicated that the higher growth rates exhibited by PB versus RB strains correlated with earlier activation of *cap5*, lower ATP levels, and higher survival rates when exposed to human-simulating VAN concentrations versus RB strains (13). Similarly, in the current studies, we demonstrated that RB 301-188p::ϕSA169, similar to the PB 300-169p strain, had significantly higher *cap5* promoter activation and higher growth rates than its parental RB 301-188p strain (Fig. 3A and B; *P *< 0.05). In addition, the 301-188p::ϕSA169 strain, as well as the 300-169p strain, exhibited significantly lower ATP levels and higher survival rates during VAN exposure than the RB 301-188p strain, even with the same VAN MICs (Fig. 3C; *P *< 0.05).

**FIG 4:**
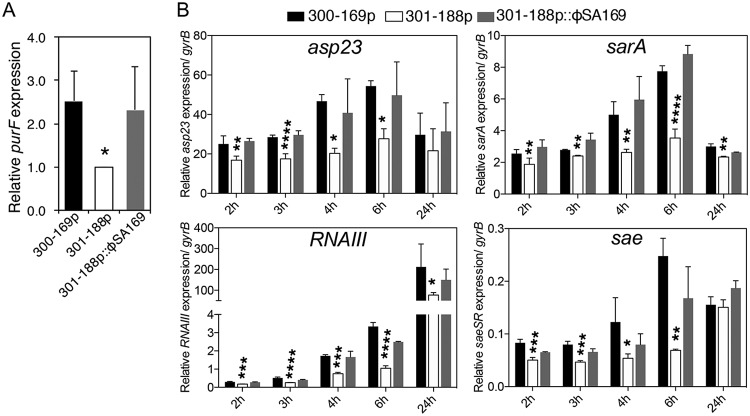
Relative expression levels of *purF* at the 3-h incubation time point (A) and *asp23* (as a surrogate for *sigB* activation), *sarA*, *RNAIII*, and *sae* at 2-, 3-, 4-, 6-, and 24-h incubation time points (B) in 300-169p, 301-188p, and 301-188p::ϕSA169 strains. *, *P* < 0.05; **, *P* < 0.01; ***, *P* < 0.001; ****, *P* < 0.0001, versus 301-188p strain.

To better understand the underlying genetic mechanisms of the faster growth of 301-188p::ϕSA169, we assessed the expression levels of *purF* in the study MRSA strains; we have previously reported that higher expression of *purF* results in higher growth rates and subsequently leads to the reduction of VAN susceptibility in PB strains (13). In this study, the 300-169p and 301-188p::ϕSA169 strains showed significantly higher *purF* expression than the strain 301-188 counterpart (Fig. 4A; >2-fold, *P *< 0.05), indicating that the faster growth in 301-188p::ϕSA169 was at least partially due to the higher *purF* expression versus 301-188p.

### The RB strain containing ϕSA169 exhibited earlier global regulator transcription profiles than its isogenic RB parental strain without ϕSA169.

Our previous studies demonstrated that PB strains exhibited significantly earlier activation of key global regulators involved in pathogenesis of S. aureus, including *asp23*, *sarA*, *agr RNAIII*, and *sae* versus RB strains (13). Importantly, temporal *agr RNAIII* expression and *agr* functional profiles have been used as biomarkers to predict the persistent outcomes in endovascular MRSA infections (5, 13, 15). In this study, we demonstrated that the transcriptions of these global regulators were higher in early-exponential-growth-phase cells in PB 300-169p and RB 301-188p::ϕSA169 strains versus the parental RB 301-188p strain (Fig. 4B).

### The RB strain containing ϕSA169 demonstrated increased biofilm formation and δ-hemolysin activities.

Biofilm formation and δ-hemolysin production (a marker of *agr* function [5]) are critical virulence factors in the pathogenesis and treatment outcomes of MRSA endovascular infections (5, 16). Similar to the PB strain 300-169p, RB strain 301-188p::ϕSA169 exhibited significantly greater biofilm formation (Fig. 3D) and enhanced δ-hemolysin production versus its isogenic strain RB 301-188p (Fig. 3E; see arrows).

### The RB strain containing ϕSA169 exhibited significantly reduced VAN treatment efficacy in the IE model.

**FIG 5:**
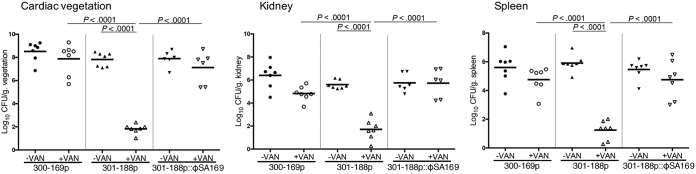
Densities of MRSA in target tissues in the IE model due to 10^5^-CFU challenges of study strains with and without VAN treatment. Each dot represents one animal. Horizontal black bars indicate mean MRSA densities.

To validate the hypothesized effect of ϕSA169 in the PB outcome *in vivo*, an experimental IE model was employed. Without VAN treatment, animals infected with 301-188p or 301-188p::ϕSA169 showed slightly lower MRSA counts (not statistically significant) in cardiac vegetation and kidney than the 300-169p counterpart (Fig. 5). Of note, similar to the PB 300-169p strain, 301-188p::ϕSA169-infected animals had no response to VAN treatment and had significantly higher MRSA densities in all target tissues than VAN-treated animals infected with the 301-188p counterpart (Fig. 5; *P *< 0.0001).

## DISCUSSION

Bacteriophages are ubiquitous in the environment and diverse in size, morphology, and genomic organization (17). Upon invading the bacterial hosts, the phage disrupts bacterial metabolism and ultimately lyses the bacteria, thus indicating its potential be an alternative treatment for human infections, especially due to antimicrobial-resistant pathogens (18). In contrast to bacterial lysis, some bacteriophage could also lysogenize and integrate into the bacterial chromosome (often called prophage) and impact bacterial evolution and virulence through toxin production, biofilm formation, and tissue adhesion (8, 9, 19). Additionally, bacteriophage is transferable and, in some cases, could cotransfer host chromosomal genes, e.g., virulence (20) and antimicrobial resistance (21) genes, even between different bacterial species (22), raising the concern about its safety in clinical usage (23).

Recent studies have identified bacteriophages as important contributors to persistent outcomes, via regulation of global virulence gene regulators and biofilm formation in other bacteria (8, 9). However, the impact of bacteriophages on persistent MRSA endovascular infections has not been previously studied. We recently demonstrated that the prototypical PB strain, 300-169, contains a unique prophage (ϕSA169) which is not present in a genetically matched RB strain, 301-188 (11). This finding led us to hypothesize that this bacteriophage might contribute to distinct PB phenotypes, particularly early-onset activation of global regulators (5, 13, 15), higher growth rates (13), reduced susceptibility to VAN (13), and greater biofilm formation (24).

In the current investigations, we first noted that viable ϕSA169 particles were observed in both recipient strains (RN4220 and RB 301-188) from not only an *in vitro* (with transduction) experiment with 300-169 (using pALC1766 as an antibiotic resistance marker for genetic transfer) but also in the plasma of 300-169-infected rabbits with IE. These results indicated that, as a typical phage, ϕSA169 can infect bacteria under both *in vitro* and *in vivo* conditions. Not surprisingly, similar results have been reported by Acheson et al. and Cornick et al., showing that viable phage virions were found in Escherichia coli strains in host gastrointestinal tract (25, 26). Meanwhile, the WGS data verified the successful transfer of bacteriophage ϕSA169 from PB 300-169 into RB 301-188, generating the 301-188::ϕSA169 strain harboring pALC1766; this further indicated that ϕSA169 is a typical “temperate” phage, which has the ability to integrate into the recipient strain’s chromosome (19). The insertion site of ϕSA169 in 301-188p::ϕSA169 is identical to the site in observed in PB 300-169. This is likely achieved by the prophage-encoded integrase (*int* gene) and the enclosing lysogeny module which processes the site-specific recombination (i.e., integration) of the phage (27). Bacteriophage-mediated spread of chromosomal genes is prevalent in both Gram-negative and Gram-positive bacteria (28), in particular, the spread of bacterial virulence genes (e.g., the S. aureus pathogenicity islands [SaPIs], frequently transferred with staphylococcal phage ϕ80α within diverse staphylococcal species) (20). Furthermore, such phage-mediated genetic transduction (e.g., SaPIs) has been documented to occur between S. aureus and Listeria monocytogenes (22). In this regard, the transduction of ϕSA169 may contribute to the distribution of bacteriophage-mediated pathogenesis factors (29).

The current study focused on the role of this novel ϕSA169 in PB signatures *in vitro* and therapeutic outcomes *in vivo*. First, the temporal gene transcription profiles revealed that all studied virulence global regulators, including *sigB*, *sarA*, *agr RNAIII*, and *sae*, activated significantly earlier in 301-188p::ϕSA169 than its isogenic parental RB strain 301-188p. This is in line with the observation by Fernández et al. that the lysogenization of a prophage activated the expression of *sigB* (9). In addition, greater δ-hemolysin production and biofilm formation were detected in 301-188p::ϕSA169 than its isogenic RB strain 301-188p, which is consistent with previously published results that key global regulators are responsible for these phenotypes (e.g., early-onset activation of *agr RNAIII* is responsible for higher δ-hemolysin production [5], and *sigB* and *sarA* regulate biofilm formation [9, 30]). Similarly, prophage ϕ11-mediated enhancement of biofilm formation was observed in S. aureus (9). Fernández et al. speculated that phage-promoted biofilm formation might be due to the increased extracellular eDNA, which is likely a consequence of cell lysis by phage (8). It has been widely investigated that bacterial prophages can spontaneously induce bacterial evolution of specific virulence factors, such as platelet-binding ability, Shiga toxin production, biofilm formation, etc. (29). Likewise, curing of all four prophages in S. aureus Newman substantially compromised its virulence (7). Interestingly, the phage gene *phi80_gp05* from ϕSA169 has also been identified in ϕ80α and ϕ11 and reported to be involved in *sigB* activation and biofilm formation in S. aureus (9). Further investigations of the ϕSA169 genes’ function, including that of *phi80_gp05*, and their contributions to the PB outcomes are needed. Taken together, ϕSA169 might contribute to the PB outcome by triggering the activation of key virulence global regulators and their respective downstream genes, which might well impact the organism’s net fitness and survival potential.

Second, we demonstrated that like the PB strain, the ϕSA169-transducted RB strain 301-188p::ϕSA169 exhibited earlier activation of *cap5* than its isogenic parental RB strain 301-188p, which suggests the RB strain containing the phage reached stationary growth phase earlier than its parental RB strain. Accordingly, significantly higher growth rates and shorter generation times were observed in PB 300-169p and 301-188p::ϕSA169 than in the parental RB strain 301-188p. These results are in line with our previous studies demonstrating that earlier activation of *cap5* positively correlates with higher growth rates (13, 14). Similar results have been observed by Bossi et al. showing that prophage could improve the fitness of Salmonella enterica serovar Typhimurium (31). The link among higher growth rates, lower ATP levels, and higher survival rates during VAN exposure in PB than in RB strains has been well established (13, 14). Indeed, this same linkage was observed in 301-188p::ϕSA169 versus its isogenic parental RB strain 301-188p. In this regard, this led us to hypothesize that reduced VAN susceptibility mediated by the prophage ϕSA169 might offer a survival advantage in the presence of VAN exposure *in vivo*.

Third, similar to the PB strain 300-169p, the 301-188p::ϕSA169 strain showed significantly higher expression levels of the purine biosynthesis pathway gene *purF* than did its isogenic parental RB strain. This genotype was reflected in faster growth in PB 300-169p and ϕSA169-transducted RB strain 301-188p::ϕSA169, as purine biosynthesis is essential for cell growth via nucleotides synthesis (32, 33). Similar results were also reported by Fernández et al., demonstrating that expression of purine biosynthesis pathway genes (e.g., *purH*) was upregulated after the lysogenization of ϕ11 or ϕIPLA-RODI in S. aureus (8, 9). In addition, Edlin et al. showed that prophages can benefit E. coli fitness under glucose-limited conditions by accelerating its metabolic activity (34). The precise mechanism(s) of prophage-mediated elevation of purine biosynthesis pathway gene expression in S. aureus is under current investigations in our laboratory.

Most importantly, the *in vivo* relevance of ϕSA169 in PB outcomes was demonstrated in the experimental IE model. Animals infected with the ϕSA169-containing RB strain 301-188p::ϕSA169, similarly to those infected with its genetically matched PB strain 300-169p, exhibited no response to VAN treatment versus animals infected with its isogenic parental RB strain. The PB outcome might be due to a combination of ϕSA169-mediated phenotypic and genotypic profiles, such as earlier onset of key global regulators and higher growth rates, subsequently enhanced biofilm formation, and reduced VAN susceptibility. Global regulators have been shown to impact transcription of key virulence genes and antimicrobial susceptibility *in vivo*, including IE (35–37). In addition, the role of *sigB* in promoting bacterial intracellular persistence by defending S. aureus from invading immune cells has been well studied (38). We, along with other investigators, have shown that *sarA* is crucial in persistent MRSA infection via its effect on the host defense system and biofilm formation, within which bacteria become more resistant to antibiotics (16, 39).

There are some important limitations to this study. Besides the remarkable PB outcomes of the lysogenized RB strain, a new question also comes along with the exciting findings: does curing prophage ϕSA169 have a reserve effect on the PB strain? Gaining prophage ϕSA169 results in acquisition of PB phenotypes; therefore, it is possible that curing ϕSA169 may ultimately lead to loss of PB phenotypes. In addition, we studied only one clinical genetic-background-matched PB-RB strain pair. It would be interesting to study if the ϕSA169 transduction in RB strains with other genetic backgrounds has a similar PB phenotype. Interestingly, we recently carried out genome analysis using 542 S. aureus genome databases published in the National Center for Biotechnology Information (NCBI), including 313 MRSA and 229 methicillin-susceptible S. aureus (MSSA) strains, by assembling nucleotide sequences of ϕSA169. This analysis was performed with online tools available on the NCBI website (https://www.ncbi.nlm.nih.gov/guide/taxonomy/) and demonstrated that there are 205 genomes (141 MRSA and 64 MSSA) showing ≥95% identity on ≥75% length of the ϕSA169 sequences. These results suggest that the ϕSA169-like sequences are present in S. aureus genomes. However, there are no data available in the NCBI database regarding what proportion of these S. aureus strains is PB versus RB. Therefore, further investigations are needed to define whether the transduction with ϕSA169 is a common and determinative event in the S. aureus strains for the PB outcome.

In summary, the present findings are, to our knowledge, the first to investigate the importance of the novel prophage ϕSA169 in the PB outcomes. Although the mechanism(s) of bacteriophage-mediated PB outcome is not well understood, these data underscore this prophage as a critical factor in persistent MRSA endovascular infections.

## MATERIALS AND METHODS

### Bacterial strains, plasmids, and growth medium.

Bacterial strains and plasmids used in this study are listed in Table 1. PB strain 300-169 was isolated from a patient with 16 days of persistent MRSA bacteremia, while RB strain 301-188 was obtained from a patient with 2 days of MRSA bacteremia (5, 15, 40). Both strains represent the initial bloodstream isolate. The two MRSA strains have a similar genetic background (e.g., CC45, *agr* I, and SCC*mec* IV) and are susceptible to VAN based upon *in vitro* CLSI breakpoints (5, 15, 40). All study strains were routinely grown at 37°C in tryptic soy broth (TSB; Difco) or on tryptic soy agar (TSA) plates if not otherwise specified.

**TABLE 1:** Staphylococcus aureus strains and plasmids used in this study

Strain or plasmid	Relevant characteristic(s)	Vancomycin MIC (μg/ml)	Reference(s)
300-169	PB^*a*^-MRSA, *agr-I* SCC*mec* IV CC45^*b*^	0.5	5
301-188	RB^*a*^-MRSA, *agr-I* SCC*mec* IV CC45	0.5	5
300-169p	300-169 with pALC1766	0.5	13
301-188p	301-188 with pALC1766	0.5	13
301-188p::ϕSA169	301-188 lysogenized with bacteriophage ϕSA169 and transduced with pALC1766	0.5	This study
RN4220	NCTC8325-4, α-hemolysin negative, β-hemolysin positive		5
SH1000	*rsbU*-positive derivative of NCTC8325-4, *agr-I*		5
SH1001	SH1000 *agr*::*tet*(M) Tet^r^		5
pALC1766	Derivative of pALC1484 in which the *cap5* promoter was cloned upstream of *gfp*_UVR_ reporter, Chl^r^		13

a PB, persistent MRSA bacteremia strain; RB, resolving MRSA bacteremia strain.

b CC, clonal complex.

### Isolation of ϕSA169 from the PB 300-169 strain.

The supernatant of stationary-phase 300-169 cells with/without MMC (1 μg/ml; Sigma; as an agent to induce the liberation of temperate phages) (41) exposure and plasma from 300-169 strain-infected rabbits in an experimental infective endocarditis (IE) model (see detailed description under “Experimental IE model” below) were filtered (0.22 μm; Millex; Millipore Corp.) to remove bacterial cells, diluted in phage buffer (42), and mixed with a recipient strain, RN4220 (a well-studied prophage-free reference S. aureus strain). The mixtures were plated on TSA plates using a well-established double-layer technique (42, 43) and incubated at 37°C overnight or until plaques developed.

### Transduction of ϕSA169 into a genetic-background-matched RB strain, 301-188.

To transduce ϕSA169 into RB strain 301-188, PB 300-169 carrying pALC1766 (this strain is named 300-169p) was employed as the donor strain (13). The plasmid pALC1766 (chloramphenicol [CHL] resistance) was used as a marker during the transduction of ϕSA169 into the recipient RB 301-188 strain for the selection of CHL-resistant colonies. Briefly, the filtered supernatant from the MMC-induced strain of 300-169p cells was mixed with the recipient strain 301-188. The mixture was then plated on TSA plates containing CHL (10 μg/ml; Sigma). After overnight incubation at 37°C, transductant colonies were collected from the culture plates for further verification (44).

To confirm whether the transduction of ϕSA169 into the 301-188 strain was successful, two pairs of primers were designed for PCR: one pair targeted the SAUSA300_1858-*int* adjunction region (F, 5′-TATGCACGATCTGTGTGGGC-3′, and R, 5′-ACATTGGTTCGCCACCTGTA-3′), while another pair targeted one of the ϕSA169 genes, *dnaC* (F, 5′-CCAATCTTTGAATTTCACATTCGC-3′, and R, 5′-AGACAGAATTGCGATAAATGCGG-3′). All PCR products were directly sequenced, and results were aligned with the genomic sequences from the GenBank nucleotide database to confirm veracity.

### Determination of VAN MICs.

VAN MICs were determined by a standard Etest method according to the manufacturer’s recommended protocols (bioMérieux, La Balme-les-Grottes, France).

### Growth curves and generation time.

To evaluate the impact of ϕSA169 on the growth rate, overnight cultures of the study MRSA strains were adjusted to a density of 1.0 McFarland standard in phosphate-buffered saline (PBS) and diluted 1:100 with 50 ml TSB in 500-ml Erlenmeyer flasks. Samples were then incubated at 37°C with shaking at 200 rpm for 24 h. Cell growth was monitored by measuring OD_600_ (13). Generation time during the exponential phase was calculated based on the growth curve (38).

### ATP levels.

ATP levels of the late-exponential cultures (6-h incubation, corresponding to the time point at which the PB 300-169 strain showed significantly higher global regulator activation than the parental RB 301-188 strain) (13) were measured using a BacTiter Glo kit (Promega) (45); results were given as ATP concentrations normalized to CFU.

### *In vitro* VAN killing assay.

A starting inoculum of ∼10^8^ CFU/ml of late-exponential MRSA cells (the same incubation time as the ATP assay above) was exposed to 15 μg/ml VAN (to mimic targeted serum trough VAN concentrations for severe MRSA infections in humans) (5) in cation-adjusted Mueller-Hinton broth for 24 h. Survival rates were expressed as the number of surviving cells divided by the initial inoculum (5, 13).

### Biofilm formation.

*In vitro* biofilm formation under static conditions was quantified as previously described (16, 40). Adhering dye (0.1% safranin) was dissolved in 30% acetic acid, and absorption was measured as OD_490_ to quantify biofilm formation (16, 40).

### Delta-hemolytic activity.

δ-Hemolysin activity was determined by perpendicular streaking of test strains with the β-hemolysis-producing S. aureus reference strain, RN4220, on 5% sheep blood TSA plates after overnight incubation at 37°C (5). The δ-hemolysis was denoted by an enhanced area of hemolysis at the intersection of RN4220 and test strain streaks (5). Strains SH1000 and SH1001 (*agr* mutant of SH1000) were used as positive and negative controls, respectively.

### RNA isolation and target gene expression by RT-qPCR.

Total RNA of the study MRSA cells from 2, 3, 4, 6, and 24 h of incubation (representing early-, mid-, late-, post-exponential, and stationary phases, respectively) was isolated by using the RNeasy kit (Qiagen) (13, 46). DNase-treated RNA (1 μg) was transcribed into cDNA. Real-time quantitative PCR (RT-qPCR) was performed using an ABI Prism 7000 instrument (Applied Biosystems) and a SYBR green PCR master kit (Applied Biosystems). Global regulators *asp23* (a surrogate for *sigB* expression), *sarA*, *agr RNAIII*, and *sae*, as well as the purine biosynthesis pathway gene *purF*, were amplified using appropriate primers as described previously (13, 35, 36, 46); *gyrB* was used to normalize the transcript quantification. Relative quantification was calculated by the threshold cycle (ΔΔ*C_T_*) method (35).

### Determination of *cap5* promoter activation by flow cytometry.

Expression of the *cap5* promoter denotes the onset of the stationary phase of growth (13, 14). The temporal activation of the *cap5* promoter in the 300-169p, 301-188p, and 301-188p::ϕSA169 strains was detected as described previously (13). Briefly, overnight MRSA cultures were 1:100 diluted into TSB and incubated overnight at 37°C with shaking at 200 rpm for 6 h. Samples were obtained at 6 h of incubation to assess the profile of *cap5* promoter activation by flow cytometry (35, 47).

### WGS.

WGS was performed with 300-169p, 301-188p, and 301-188p::ϕSA169 strains. Genomic DNA extraction and library preparation were conducted as described previously (48, 49). The published genome JASK00000000 (301-188) was used for DNA library mapping, and SNPs and indels were evaluated (11, 50).

### Experimental IE model.

A well-characterized rabbit model of catheter-induced aortic valve IE was used to define the potential role of ϕSA169 in persistent *in vivo* outcomes during VAN treatment (5, 13). At 24 h after aortic catheterization, animals were infected intravenously with one of the following strains: 300-169p, 301-188p, or 301-188p::ϕSA169. For infection, we used 10^5^ CFU/animal, a 95% infective dose (ID_95_) dose previously established for these strains (5). Twenty-four hours after infection, animals were randomly assigned to receive either no therapy (control group) or VAN at 15 mg/kg of body weight intravenously twice daily for 3 days. Control animals were sacrificed at 24 h postinfection (starting time point for VAN treatment). VAN-treated animals were euthanized 24 h after the last treatment to avoid VAN carryover effects. The cardiac vegetation, kidneys, and spleen were removed and quantitatively cultured (5, 13). MRSA counts in the target tissues were calculated as the mean log_10_CFU/g of tissue (± standard deviation [SD]). Rabbits were cared for in accordance with the American Association for Accreditation of Laboratory Animal Care criteria. The Institutional Animal Care and Use Committee (IACUC) of the Lundquist Institute at Harbor-UCLA Medical Center approved all animal studies.

### Statistical analysis.

All *in vitro* experiments were performed in triplicate and repeated at least twice. The two-tailed Student *t* test was employed to analyze the *in vitro* data, as well as differences in tissue MRSA counts in the study groups. *P* values of <0.05 were considered statistically significant.

### Data availability.

All data supporting the findings of this study are available either within the article or in the supplemental material.
