# Supplementary figures and images for: Impact of the Novel Prophage ϕSA169 on Persistent Methicillin-Resistant Staphylococcus aureus Endovascular Infection
Source: mSystems. 2020 Jun 30;5(3):e00178-20. doi: 10.1128/mSystems.00178-20 (PMC7329321; doi:10.1128/mSystems.00178-20)

**
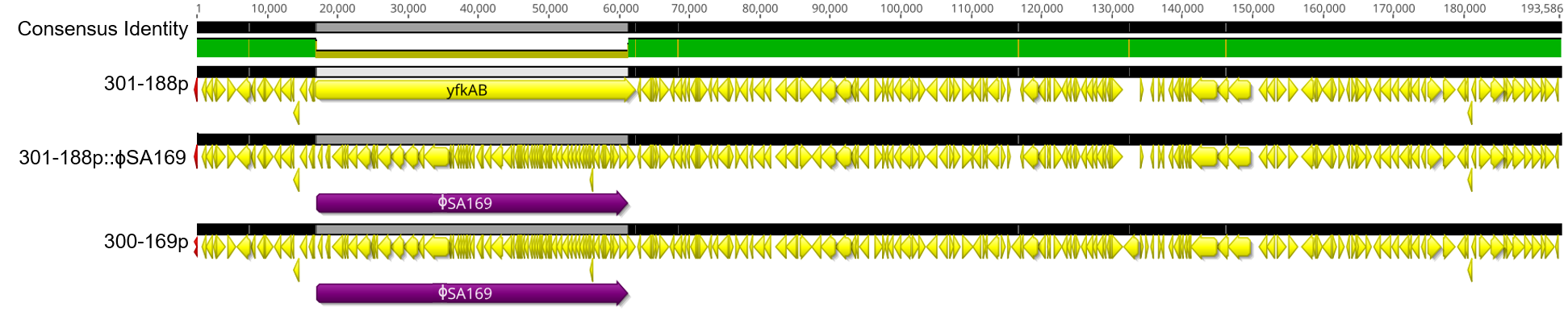
**

Supplement: FIG S1 [file mSystems.00178-20-sf001.docx]
