# Supplementary material for: Impact of the Novel Prophage ϕSA169 on Persistent Methicillin-Resistant Staphylococcus aureus Endovascular Infection
Source: mSystems. 2020 Jun 30;5(3):e00178-20. doi: 10.1128/mSystems.00178-20 (PMC7329321; doi:10.1128/mSystems.00178-20)
Supplement: TABLE S1 [file mSystems.00178-20-st001.docx]

| References  (Genbank accession No.) | Position | 301-188 | 301-188p::ɸSA169 | 301-188p | Product |
| --- | --- | --- | --- | --- | --- |
| JASK01000001 | 310759 | C | G | C | Hypothetic protein |
| JASK01000004 | 448486 | A | A | G | Siderophore biosynthesis protein |
| JASK01000005 | 9318 | C | T | T | phosphoenolpyruvate carboxykinase |
